# Building Curriculum Vitae as a Medical Student

**DOI:** 10.31729/jnma.8019

**Published:** 2023-02-28

**Authors:** Saroj GC, Reshika Shrestha

**Affiliations:** 1 Maharajgunj Medical Campus, Maharajgunj, Kathmandu, Nepal

**Keywords:** *career*, *hobbies*, *leadership*, *medical students*, *research*

## Abstract

The curriculum vitae is the standard means of communication of accomplishments that are relevant to the academic realm. The purpose of this is to provide a brief, digestible summary of one's personal and professional life. The quality of a curriculum vitae is more important than the quantity, and it takes skill to write one that is organized, clear, and brief. From their first year of medical school, medical students can get involved in research and publishing, plan activities that develop their leadership and management abilities, pursue their interests, and attend national and international conferences. At the end, it's all about working on yourself and developing a distinct professional and personal identity which can be effectively reflected in your curriculum vitae.

## INTRODUCTION

The curriculum vitae (CV) is a written document where we trade our achievements and personality traits in search of opportunities.^1^ One of the vital things we as a student tend to realise later is working on a CV thus in the end we only have our academic part highlighted in them. But a CV needs to be wholesome as there are other equally important aspects which need to be covered to make a CV that stands out. A CV should be concise, easy to read and summarise important aspects of one's professional and personal history.^1^

## IMPORTANCE OF CV

It is a comprehensive document with your personal information, skills, educational and professional qualifications and achievements imparting a sense of professionalism, competence, and enthusiasm.^2^ A CV has historically been a prerequisite for applying for any national or international opportunity, including residency abroad. It's a crucial step in the application process for residency in various nations because it's the first thing most selection committee members can look at to know about applicants' accomplishments and separate them from the rejection piles.^3^ CV has been considered as a shop-window, it about selling yourself. A distinctive, well-written, and personalized CV will set apart stellar from other candidates during the selection process in today's fiercely competitive world. One should therefore assume that this may be the recruiters' first and only unforgettable impression of them.^4^

## BUILDING DIFFERENT ASPECTS OF CV

**1. Management and leadership experience**

It might be beneficial for medical students to join organizations and clubs in medical colleges and outside the college which are established with specific purposes. Medical students can start doing volunteering activities from the initial years. They can manage time during summer or winter breaks or during short holidays. They can involve in and organize events like sports activities, training, campaigns, advocacy efforts, health awareness projects, health camps, conferences, workshops, medical education programs, mentoring juniors, etc.

Some organizations are affiliated with international bodies so these activities will provide a network that links medical students in international platforms to present their work, and expose them to humanitarian and health issues. They can involve in different organizations and build up professionalism, leadership abilities and communication skills during medical school.

If feasible medical students can create their own event, organisations or opportunities during medical school. For these, its always better to be proactive and explore these opportunities during early life. These soft skills will shape attitude and behaviour in the personal and professional life.^2^

**2. Research and publications experience**

For medical students, it is better to do a simple and short project which can be done with our resources and will get finished by the time of graduation. Be proactive, find a good mentor and involve in the one in which you think you are going to learn about the research process. You can start from the research section which is specifically targeted and focused on medical students like Student BMJ, Student JNMA, etc. They can read articles about the topic of interest from the high-impact factor journals to be acquainted with the recent advances and research. Medical students can write medical and surgical Case Reports under the supervision and guidance of treating consultants and resident doctors. Medical students can assist and involve in research projects by teachers or can do their own research of simpler study design which needs a short time, less resources and less expertise which can be managed on our own.

Before starting research projects, medical students can take training programs and research workshops from academic institutions either online or in person. We should be aware of the trainer whether he or she had an academic degree in research-based courses like epidemiology, medical statistics, clinical trial, clinical research, systematic review and metanalysis, etc. Not all clinical experts and those with dozens of publications might not be good sources for learning research skills. We should accept the limitations of resources in our setting and expertise and be involved in those projects which are most feasible and by doing so we can develop basic research skills.

Peer-reviewed and PubMed-indexed journals might shine a light upon a student's merit and worth. Publications represent students' hard work and aptitude, consistency, continuous dedication, and intellectual capabilities. Involving in research and publication during medical school, not only boosts your CV but research skills help to continue to do research, and improve your critical appraisal of the literature, and in-depth knowledge of the topic of interest.^5^

**3. Other publications (Book, magazine, online portal)**

Medical students can mention if they have published any books, or writings in magazines and in any online portal.

**4. Participation in conferences, workshops and development courses**

Medical students can participate in conferences on different topics or subject specialities, research and training workshops, and other academic development courses. These mentions are definitely a boost to our CV.

**5. Oral presentations, poster presentations in conferences**

Medical students can also present their research work through oral presentations using PowerPoint slideshow or in the form of posters using visual representation through text, charts, graphs, and other visual aids. Participating in virtual and in-person presentations, will be helpful in finding different ways to clarify the same concept and analyse which explanation is most accepted by the audience. This will also help hone their communication skills and improve ability to present information more simply^6^

**6. Personal interests and hobbies**

Hobbies are activities that you enjoy doing and are not the responsibilities of school or work. Hobbies makes evey individual unique. These activities will reflect your personalities like teamwork, learning attitude, time management, and responsibility. Hobbies reflect your values, character and ways of thinking that will help you develop as a person and professional.

Commonly mentioned hobbies by medical students include sports (football, basketball, cricket, table tennis, etc), arts (painting, writing, drawing, playing musical instruments), running, swimming, hiking, biking, travelling, photography, cooking/baking, content creation, etc. For the interviewer, these will help to know how you will cope the stressful situations and show a balanced approach to personal and professional life.^7^

There is always time constraint during medical school so medical students tend to sacrifice their their hobbies. But if we make our daily schedules wisely, and are dedicated enough then surely we can adjust our hobbie time in our daily life. Moreover, we could utilise our weekends, vacations, and even long exam study gaps. Dashain vacation, and Summer/Winter vacations (in initial medical years) could be managed for travelling. So, we should be proactive and continue our hobbies alongside studies.

**7. Teaching-learning experiences**

Teaching-learning is an intrinsic part of a medical career. One day medical students will be mentors or supervisors and will be teaching juniors or students on regular basis. Teaching can vary in setting and audiences and can be one-to-one, in a group or in a large scale. So, it is one of the important skills for a medical student.

**8. Information technology skills**

Digital documentation for maintaining patient records will be available in all the hospitals in the future so it is advisable to have effective typing and other basic computer skills for all medical students. Gadgets can be used for maintaining daily schedule, appointments, study log, exercise log, etc and can become more effective and efficient in daily life. During leisure time, medical students can learn basic to advanced computer skills that are commonly used like MS Excel, MS Word, MS PowerPoint, google apps etc. Medical students can also learn statistical software packages like IBM SPSS Statistics, R-programming, STATA, etc.

## STRATEGIES FOR WRITING CV

During the preparation phase, we should go through as many CVs as possible for reference. But while making one, the CV needs to be clear, concise and relevant.^1^ A smart medical student starts working on their CV from the first year of medical school and keeps updating any achievements accomplished henceforth. Having the CV updated and ready always come in handy. But during the process, we need to maintain honesty and refrain from fabricating information. Also, when stating the credentials, it would be wise to avoid sounding self-congratulatory.^1,2^

Reverse chronological order must be adhered to for all entries. The use of different fonts and formatting in each area of the CV should be avoided. The CV's structure should be consistent in its layout, space, etc. Editing should be done to ensure proper language, spelling, and punctuation. Subsections should be presented as bullet points rather than paragraphs.^2^

After completion, we can set it aside for a while, then go back and update it with new information later. This final draft can be given to a professional mentor or friend as well as to those with experience reviewing materials of this nature.^3^ If you have a printed copy of your resume, make sure you use high-quality paper, ink, and tools. Only print on one side and make sure there are no smudges or streaks on any of the sheets. To secure the pages, affix a single staple to the upper-left corner of each page. Additionally, the candidate ought to have extra copies on hand for use in interviews.

## WAY FORWARD

As medical students, we need to start early on working on our CV. We need to take time to reflect on our values, skill sets and what we want next for our careers. The CV needs to get updated with time and our achievements. During our medical student years, one should grab every opportunity and engage in educational and extracurricular activities.
